# An Intellectual Disability-Related Missense Mutation in Rac1 Prevents LTP Induction

**DOI:** 10.3389/fnmol.2018.00223

**Published:** 2018-07-06

**Authors:** Chen Tian, Yuni Kay, Anastasiia Sadybekov, Sadhna Rao, Vsevolod Katritch, Bruce E. Herring

**Affiliations:** ^1^Department of Biological Sciences, University of Southern California, Los Angeles, CA, United States; ^2^Department of Chemistry, University of Southern California, Los Angeles, CA, United States; ^3^The Bridge Institute, University of Southern California, Los Angeles, CA, United States

**Keywords:** intellectual disability, glutamatergic synapse, long-term potentiation (LTP), Rac1, GTP binding, AMPA receptor

## Abstract

The small GTPase Rac1 promotes actin polymerization and plays a critical and increasingly appreciated role in the development and plasticity of glutamatergic synapses. Growing evidence suggests that disruption of the Rac1 signaling pathway at glutamatergic synapses contributes to Autism Spectrum Disorder/intellectual disability (ASD/ID)-related behaviors seen in animal models of ASD/ID. Rac1 has also been proposed as a strong candidate of convergence for many factors implicated in the development of ASD/ID. However, the effects of ASD/ID-related mutations in Rac1 itself have not been explored in neurons. Here, we investigate a recently reported *de novo* missense mutation in Rac1 found in an individual with severe ID. Our modeling predicts that this mutation will strongly inhibit Rac1 activation by occluding Rac1’s GTP binding pocket. Indeed, we find that this *de novo* mutation prevents Rac1 function and results in a selective reduction in synaptic AMPA receptor function. Furthermore, this mutation prevents the induction of long-term potentiation (LTP), the cellular mechanism underlying learning and memory formation. Together, our findings strongly suggest that this mutation contributes to the development of ID in this individual. This research demonstrates the importance of Rac1 in synaptic function and plasticity and contributes to a growing body of evidence pointing to dysregulation of actin polymerization at glutamatergic synapses as a contributing factor to ASD/ID.

## Introduction

Approximately 1%–3% of the general population is affected by intellectual disability (ID). ID is a neurodevelopmental disorder defined by significant limitations in intellectual functioning and adaptive behavior and is often comorbid with autism spectrum disorders (ASD; Mefford et al., 2012). Human and animal ASD/ID model research has converged on altered glutamatergic synaptic function as a potential cause of cognitive dysfunction (Bagni and Greenough, 2005; Zoghbi and Bear, 2012; Volk et al., 2014). Synapses allow communication between neurons and are essential for learning and memory formation in the brain. Learning and memory formation rely on long lasting increases in glutamatergic synapse strength produced by the cellular process of long-term potentiation (LTP). Synaptic strength is largely influenced by changes in synaptic structure (Matsuzaki et al., 2004; Herring and Nicoll, 2016a), and synaptic structure is dictated by regulation of the synaptic actin-cytoskeleton. Rho GTPases are key regulators of actin polymerization and have been generally implicated in ASD/ID (Pavlowsky et al., 2012). Disruption of the small Rho GTPase Rac1, in particular, has been identified in common ASD/ID animal models, and Rac1 has been proposed as a convergence point for many ASD/ID genes (Schenck et al., 2003; Dolan et al., 2013; Zeidán-Chuliá et al., 2013; Duffney et al., 2015).

Rac1 orchestrates synaptic actin polymerization and is essential for synaptic function and plasticity (Tashiro et al., 2000; Wiens et al., 2005; Martinez and Tejada-Simon, 2011). Rac1 switches between an active GTP-bound state and an inactive GDP-bound state, and Rac1’s activity is tightly regulated by protein activators (GEFs) and inhibitors (GAPs). Guanine nucleotide exchange factors, or GEF proteins, activate Rac1 by exchanging GDP for GTP while GTPase accelerating proteins, or GAP proteins, induce GTP hydrolysis. In a previous study, we discovered that Rac1 GEFs, Kalirin and Trio, are essential for the induction of LTP (Herring and Nicoll, 2016b). Furthermore, we have found an ASD-related *de novo* mutation hotspot in Trio’s Rac1 activating domain. These ASD-related mutations in Trio bidirectionally alter Rac1 activation, leading to either abnormally weak or strong glutamatergic synapses (Sadybekov et al., 2017). However, the influence of ASD/ID related mutations in Rac1 itself has not been explored in neurons.

**Figure 1:**
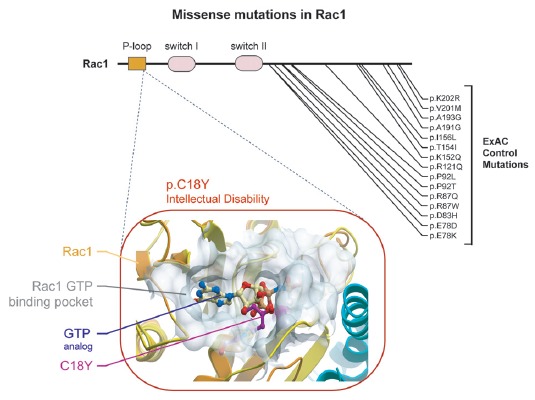
A *de novo* missense mutation in the P-loop region of Rac1 in an individual with severe intellectual disability (ID) is predicted to prevent Rac1 activation. (Top) Rac1 protein regions are indicated, starting with the N terminus: P-loop region, switch I and switch II. Location of a *de novo* ID-related missense mutation and control missense mutations are shown. (Bottom) The predicted effect of the severe ID-related mutation is shown. Rac1’s ribbon structure is shown in orange (Protein Data Bank code 3TH5). Rac1’s GTP binding pocket is shown as a transparent white surface. The tyrosine at position 18 observed in an individual with severe ID is labeled and shown in stick representation and colored magenta. A GTP analog (GMP-PNP) is shown in stick representation with carbon atoms colored pale yellow.

Recently, analysis of 2104 patient-parent trios among multiple ID studies identified 10 new ID related genes (Lelieveld et al., 2016). Among these genes was *RAC1*. In this exome sequencing study, an individual with severe ID was found harboring a *de novo* mutation in the *RAC1* gene that changes the cysteine in position 18 of the Rac1 protein (C18) to a tyrosine (C18Y; Figure 1). Here, we characterize the impact of this recently reported Rac1 ID-related *de novo* missense C18Y mutation on synaptic function. Computational modeling, electrophysiological recording and super resolution imaging demonstrate that this severe ID-related mutation occludes Rac1’s GTP binding pocket and disrupts the function of glutamatergic synapses. Furthermore, we find that the expression of this severe ID-related Rac1 mutation blocks LTP, the cellular basis of learning and memory formation. Our results suggest that the ID observed in this individual stems from altered Rac1 function and the resulting synaptic dysfunction. This study supports synaptic Rac1-mediated actin regulation as a key convergence point of molecular perturbations previously associated with ASD/ID.

## Materials and Methods

### Electrophysiology

#### Organotypic Hippocampal Slices

400 μm organotypic hippocampal slice cultures were prepared from P6 to P9 Sprague-Dawley rat pups as described previously (Stoppini et al., 1991). Culture media was exchanged every other day. Sparse biolistic transfections of organotypic slice cultures were carried out on DIV4 or DIV12 as described (Schnell et al., 2002). Construct expression was confirmed by GFP and mCherry co-transfection. Paired whole-cell recordings from transfected neurons and non-transfected control neurons were performed on DIV7 or DIV15 slices. During recording, all slices were maintained in room temperature artificial cerebrospinal fluid (aCSF) saturated with 95% O_2_/5% CO_2_. aCSF contained 119 mM NaCl, 2.5 mM KCl, 1 mM NaH_2_PO_4_, 26.2 mM NaHCO_3_, 11 mM glucose, 4 mM CaCl_2_, 4 mM MgSO_4_, supplemented with 5 μM 2-chloroadenosine to dampen epileptiform activity and 0.1 mM picrotoxin to block GABA(A) receptors. The internal whole-cell recording solution contained: 135 mM CsMeSO_4_, 8 mM NaCl, 10 mM HEPES, 0.3 mM EGTA, 5 mM QX-314, 4 mM Mg-ATP, and 0.3 mM Na-GTP. The internal solution was pH buffered at 7.3–7.4 and osmolarity was adjusted to 290–295 mOsm. Whole-cell recordings were carried out as described (Sadybekov et al., 2017). Synaptic responses were evoked by stimulating with a monopolar glass electrode filled with aCSF in stratum radiatum of CA1. AMPA receptor-mediated currents were measured at −70 mV. NMDA receptor-mediated currents were recorded at +40 mV, temporally isolated from AMPAR currents by measuring amplitudes 250 ms following the stimulus. In most cases AMPAR and NMDAR mediated currents were recorded from the same neuron by changing the membrane potential. In the scatterplot, each open circle represents one paired recording, with the y-axis value for transfected neuron eEPSC amplitude and the x-axis value for control neuron eEPSC amplitude. When eEPSC amplitudes in control neurons are higher than that of transfected neurons, data points fall below the diagonal line. When eEPSC amplitudes in control neurons are lower than that of transfected neurons, data points fall above the diagonal line. Paired-pulse ratio was recorded by delivering two stimuli at intervals of 20 ms, 40 ms, 70 ms and 100 ms and dividing the peak response of stimulus two by the peak response of stimulus one. 0.1 mM spermine was added to intracellular solution described above for measurement of AMPA receptor-mediated current rectification. Rectification indices were calculated as the normalized glutamate-evoked current at +40 mV over −70 mV, respectively, in presence of 100 μM APV to block NMDAR-mediated EPSCs. This calculation was as follows: RI = 7(I_40_ – I_0_)/4(I_0_ – I_70_) where I_x_ represent EPSC amplitude at x mV. A built-in single exponential decay fit function in IgoR was used to calculate decay time constants for AMPAR-eEPSCs. No more than one paired recording was performed on a given slice. This study was carried out in accordance with the National Institutes of Health (NIH) Guide for the Care and Use of Laboratory Animals and the protocol was approved by the University of Southern California Institutional Animal Care and Use Committee.

#### Acute Hippocampal Slices

Mice were electroporated on E15 as previously described in Herring and Nicoll (2016b). 300 μm acute hippocampal slices from P21 to P29 mice were prepared using D.S.K microslicer ZERO1 vibrating microtome (Ted Pella, CA, USA) in high sucrose low sodium ice-cold cutting solution saturated with 95% O_2_/5% CO_2_. Cutting solution contained 2.5 mM KCl, 0.5 mM CaCl_2_, 7 mM MgCl_2_, 1.25 mM NaH_2_PO_4_, 25 mM NaHCO_3_, 7 mM glucose, 210 mM sucrose and 1.3 mM ascorbic acid. After cutting, slices were incubated in aCSF at 37°C for at least 40 mins and at room temperature for another 40 mins before recording.

Slices were transferred to a submersion chamber for recording and maintained in room temperature aCSF saturated with 95% O_2_/5% CO_2_. aCSF contained 119 mM NaCl, 2.5 mM KCl, 1 mM NaH_2_PO_4_, 26.2mM NaHCO_3_, 11 mM glucose, 2.5 mM CaCl_2_, 1.3 mM MgSO_4_, supplemented with 0.1 mM picrotoxin to block GABA(A) receptors. Internal solution was identical to that used with organotypic slice culture recording (see above). Synaptic responses were evoked by stimulating with a monopolar glass electrode filled with aCSF in stratum radiatum of CA1. AMPAR currents were measured at −70 mV. A “pairing” stimulation protocol was used to induce LTP. Our pairing LTP induction protocol consisted of a single train of 2 Hz Schaffer collateral stimulation for 90 s while holding the postsynaptic neuron at 0 mV. This induction protocol was applied within 5 mins of achieving whole-cell configuration to avoid “wash-out” of LTP. Individual experiments were normalized to the baseline before stimulation and 12 consecutive responses were averaged to generate 1-min bins, which were then averaged to generate summary graphs. Bar graphs of LTP magnitudes were produced based on the averaged eEPSC values for the first 2 mins (prior to LTP induction) and last 2 mins of LTP summary graphs.

### Coefficient of Variation Analysis

The locus of alterations of eEPSC amplitude was estimated by comparing the change in eEPSC variance with the change in mean amplitude (Bekkers and Stevens, 1990; Malinow and Tsien, 1990; Gray et al., 2011). The coefficient of variation (CV) was calculated as SD/M, where M and SD are the mean and standard deviation of eEPSC amplitude, respectively. The M and SD were measured for a concurrent set of stimuli (25–60 sweeps per pair) from a control and neighboring transfected cell. Pairs with less than 25 sweeps were excluded from CV analysis. It has been shown theoretically and experimentally that changes in CV^−2^ (M^2^/SD^2^) are independent of quantal size but vary in a predictable manner with quantal content: number of release sites *n* × presynaptic release probability, Pr; CV^−2^ = *n*Pr/(1 − Pr) (del Castillo and Katz, 1954; Bekkers and Stevens, 1990; Malinow and Tsien, 1990; Xiang et al., 1994). CV analysis is presented as scatterplots with CV^−2^ values calculated for transfected cell/control cell pairs on the *y*-axis and mean eEPSC amplitude values of transfected cell/control cell pairs on the *x*-axis. Filled circles represent the mean ± SEM of the entire dataset. Filled circles that fall on or near the 45° (*y* = *x*) line suggest changes in quantal content while values approaching the horizontal line (*y* = 1) suggest a change in quantal size. Unsilencing of synapses can mimic an increase in the number of release sites when presynaptic release probability is unchanged (for discussion see Kerchner and Nicoll, 2008). Linear regressions were obtained using the least squares method.

### Spine Density Analysis

Control and experimental CA1 pyramidal neurons in cultured hippocampal slice prepared from P6 to P8 rat pups were biolistically transfected with pFUGW-GFP and pCAGGS-IRES-mCherry constructs on DIV4. Images were acquired by experimenter, blinded to condition, on DIV7 using super-resolution microscopy (Elyra Microscope System, Zeiss). For use with the inverted microscope and oil-immersion 100× objective lens, slices were fixed in 4% PFA/4% sucrose in PBS, washed 3× with PBS and cleared with an abbreviated SeeDB-based protocol (Ke et al., 2013). Image acquirement and analysis were carried out as described previously (Sadybekov et al., 2017).

### Modeling

The effect of mutations on GTP binding were predicted using the high-resolution structure of Rac1 in complex with the slowly hydrolyzing GTP analog guanosine-5′-(βγ-imido) triphosphate (GMP-PNP; PDB code 3TH5). Calculations were performed using ICM molecular modeling software (Molsoft LLC).

### Experiment Constructs

Human Rac1 and Rac1b cDNA were purchased from Genescript (clone ID OHu23004D for Rac1 and OHu22224C for Rac1b) and cloned into a pCAGGS vector containing IRES-mCherry. ID-related mutations were made from Rac1 and Rac1b cDNA using overlap-extension PCR followed by in-fusion cloning (Clontech). All plasmids were confirmed by DNA sequencing. A pFUGW vector expressing only GFP was co-expressed with pCAGGS-IRES-mCherry constructs to enhance identification of transfected neurons and was used as a control vector for spine imaging.

## Results

### A Severe ID-Related *de Novo* Mutation Is Predicted to Prevent Rac1 Activation

A recent exome sequencing study identified *RAC1* as a novel ID risk gene (Lelieveld et al., 2016). This study identified an individual with severe ID that harbored a *de novo* missense mutation resulting in the substitution of a cysteine residue for a tyrosine at position 18 (C18Y) of Rac1’s amino acid sequence. This residue is inside the P-loop region of Rac1. Specifically, this residue resides within Rac1’s GTP binding pocket. Previous structural analysis suggests that Rac1 C18 interacts directly with GTP (Hirshberg et al., 1997; Worthylake et al., 2000). Together, this evidence suggests that C18 plays a role in Rac1 function. We found no mutations in Rac1’s P-loop in the ExAC control genome database (Lek et al., 2016; Figure 1), which accentuated the importance of residue C18. We then used a structure-based modeling approach to predict the effect of this ID-related mutation on Rac1 function. Our modeling of the C18Y mutation showed that replacing the cysteine at this position with a tyrosine causes the aromatic R group of the tyrosine residue to extend into the middle of the Rac1’s GTP binding pocket. Thus, this ID-related mutation is likely to disrupt GTP-mediated activation of Rac1 (Figure 1).

### Rac1 C18Y Inhibits Synaptic Function

**Figure 2:**
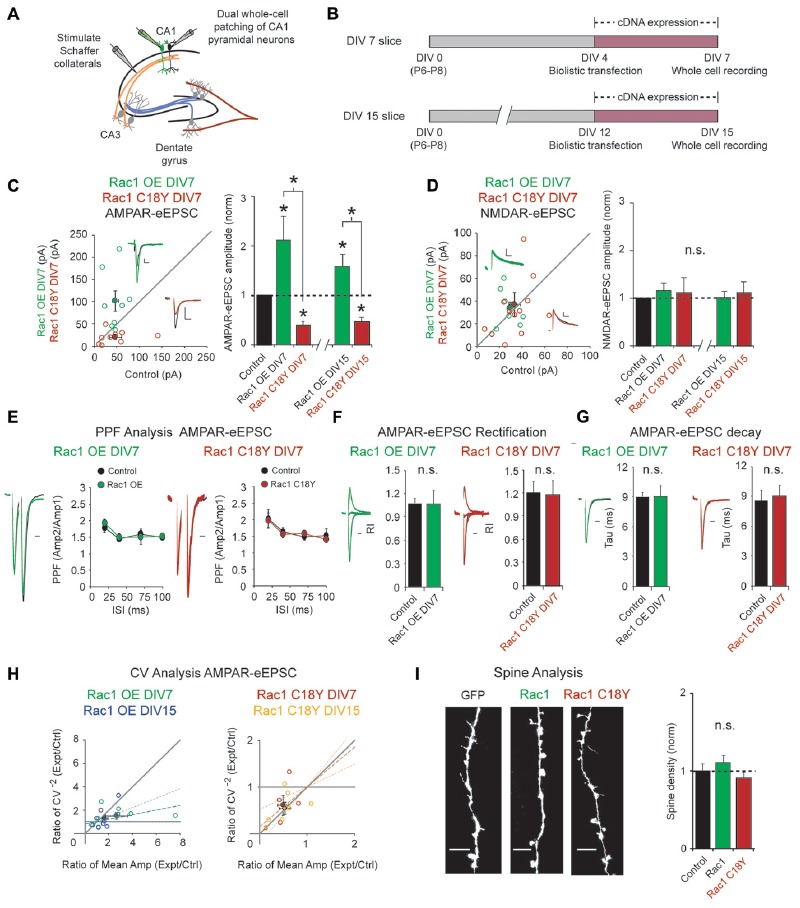
Rac1 C18Y weakens glutamatergic synaptic transmission. **(A)** Electrophysiology recording setup. **(B)** Timeline of transfection and recording. **(C,D)** Scatterplots show eEPSC amplitudes recorded at DIV7 for single pairs of CA1 pyramidal neurons transfected with Rac1 (green) or Rac1 C18Y (red) and their corresponding control neurons (*open circles*). Filled circles show mean ± SEM (*insets*). Current traces from control (*black*) and transfected (*green for Rac1, red for Rac*1 C*18Y*) neurons are shown (*Scale* bars: 20 ms for AMPA, 50 ms for NMDA, 20 pA). Bar graphs show the average eEPSC amplitudes (± SEM) of neurons expressing Rac1 or Rac1 C18Y normalized to their respective average control eEPSC amplitudes at DIV7 and DIV15. Wilcoxon Rank Sum Tests were used to compare across independent conditions (i.e., Rac1 and Rac1 C18Y in C, *P < 0.05). **(C)** Rac1 expression increased AMPAR-eEPSC amplitude in DIV7 (*n* = 8 pairs, *P < 0.05) and DIV15 slices (*n* = 9 pairs, *P < 0.05). Rac1 C18Y expression reduced AMPAR-eEPSC amplitude in DIV7 (*n* = 9 pairs, *P < 0.05) and DIV15 slices (*n* = 7 pairs, *P < 0.05). Significance was determined by Wilcoxon Signed Rank Tests. **(D)** Rac1 and Rac1 C18Y expression did not affect NMDAR-eEPSC amplitude in DIV7 (Rac1: *n* = 9 pairs, *P* > 0.05, Rac1 C18Y: *n* = 12 pairs, *P* > 0.05, Wilcoxon Signed Rank Test) or in DIV15 slices (Rac1: *n* = 9 pairs, *P* > 0.05, Rac1 C18Y: *n* = 7 pairs, *P* > 0.05, Wilcoxon Signed Rank Test). n.s., not significant. **(E)** Rac1 and Rac1 C18Y expression did not affect paired-pulse facilitation (PPF) ratios at interstimulus intervals of 20, 40, 70 and 100 ms (Left plot: 20 ms: *n* = 7 pairs, 40 ms: *n* = 5 pairs, 70 and 100 ms: *n* = 6 pairs, *P* > 0.05, Student’s *t*-test; Right plot: 20 ms: *n* = 7 pairs, 40 ms: *n* = 8 pairs, 70 ms: *n* = 5 pairs, 100 ms: *n* = 6 pairs, P > 0.05, Student’s *t*-test). Peak 1-scaled current traces from control (*black*) and transfected (*green for Rac1, red for Rac1 C18Y*) neurons are shown. (Scale bar: 20 ms). n.s., not significant. **(F)** Rac1 and Rac1 C18Y expression did not change AMPAR-eEPSC rectification. Bar graphs show mean ± SEM of the AMPAR-eEPSC rectification index recorded in the presence of AP5. (Left graph: control: *n* = 5, Rac1: *n* = 5, *p* > 0.05, Wilcoxon Signed Rank Test; Right graph: control: *n* = 7, Rac1 C18Y: *n* = 6, *p* > 0.05, Wilcoxon Signed Rank Test). Representative traces (*green for Rac1, red for Rac1 C18Y*) are shown to the left of graph (Scale bars: 20 ms). **(G)** Rac1 and Rac1 C18Y expression did not affect AMPAR-eEPSC decay. Bar graphs show mean ± SEM of the AMPAR-eEPSC decay kinetics. (Left graph: *n* = 8, *p* > 0.05, Wilcoxon Signed Rank Test; Right graph: *n* = 7, *p* > 0.05, Wilcoxon Signed Rank Test). Representative traces (*green for Rac1, red for Rac1 C18Y*) are shown to the left of graphs (Scale bars: 20 ms). **(H)** Coefficient of variation (CV) analysis of AMPAR-eEPSCs from pairs of control and Rac1/Rac1 C18Y neurons at DIV7 and DIV15. CV^−2^ ratios are graphed against the mean amplitude ratio for each pair (*open circles*; *green for Rac1 DIV7*: *n* = 7 pairs; *blue for Rac1 DIV15*: *n* = 9 pairs; *red for Rac1 C18Y DIV7*: *n* = 5 pairs; *yellow for Rac1 C18Y DIV15*: *n* = 7 pairs). Filled circles show mean ± SEM. Dashed lines show linear regression and 95% confidence intervals. **(I)** Rac1 and Rac1 C18Y expression did not affect spine density. Representative dendritic spine images from neurons transfected with GFP (control), Rac1 or Rac1b C18Y are shown on the left (Scale bars: 5 μm). The bar graph shows average spine density (mean ± SEM) of neurons expressing Rac1 or Rac1 C18Y normalized to GFP expressing control neurons (control: 0.28 ± 0.024 spines/μM, *n* = 8, Rac1: 0.31 ± 0.024 spines/μM, *n* = 11, Rac1 C18Y: 0.26 ± 0.021 spines/μM, *n* = 16, *P* > 0.05, Student’s *t*-test).

Regulation of glutamatergic synapse strength is critical for information storage in the brain, and Rac1 is a key regulator of glutamatergic synapses. To examine the impact of this ID-related Rac1 mutation on glutamatergic neurotransmission, we used biolistic transfection to express wild-type Rac1 or Rac1 C18Y in CA1 pyramidal neurons of rat organotypic hippocampal slice cultures. Given that ID is a neurodevelopmental disorder, we reasoned that Rac1 C18Y may impact synaptic development. To test this, we transfected CA1 pyramidal neurons in DIV4 hippocampal cultures with either Rac1 or Rac1 C18Y constructs. Three days after transfection, we recorded AMPA receptor and NMDA receptor-evoked excitatory postsynaptic currents (AMPAR and NMDAR-eEPSCs) following Schaffer collateral stimulation from transfected fluorescent neurons and neighboring untransfected control neurons, simultaneously (Figures 2A,B). This approach permits a pair-wise, internally controlled comparison of the consequences of the genetic manipulation. We found that Rac1 overexpression produced a 2-fold increase in AMPAR-eEPSC amplitude (Figure 2C). In marked contrast to wild-type Rac1, expression of Rac1 C18Y led to a 60% decrease in AMPAR-eEPSC amplitude (Figure 2C). The alterations of synaptic function produced by Rac1 and Rac1 C18Y were selective for AMPAR-eEPSC amplitude, with no change observed in NMDAR-eEPSC amplitudes (Figure 2D). Together, these data demonstrate Rac1’s effect on glutamatergic synapses is severely altered by the C18Y mutation, and that Rac1 C18Y produces an inhibitory effect on AMPAR-mediated synaptic transmission. To determine if specific time points in development are sensitive to Rac1 C18Y expression, we expressed either Rac1 or Rac1 C18Y in older CA1 pyramidal neurons in DIV12 cultures and recorded from these neurons 3 days later at DIV15 (Figure 2B). Expression of Rac1 and Rac1 C18Y in DIV15 cultures produced effects on synaptic transmission that were very similar to DIV7 cultures (Figures 2C,D; Supplementary Figures S1A,B). We then examined whether the alterations in AMPAR-eEPSC amplitude we observed occur as a result of changes in presynaptic glutamate release by evaluating paired-pulse facilitation (PPF). We found that neither neurons transfected with Rac1 nor neurons transfected with Rac1 C18Y exhibited any change in PPF compared to control neurons over a range of paired stimulation intervals. These data demonstrate that Rac1 and Rac1 C18Y-mediated alterations in AMPAR-eEPSC amplitude do not result from alterations in presynaptic glutamate release (Figure 2E; Supplementary Figure S1C).

Given that AMPAR subunit composition affects AMPAR function, we asked whether subunit composition of postsynaptic AMPARs is altered by either overexpression of wild-type Rac1 or expression of Rac1 C18Y. AMPARs are composed of heterotetrameric assemblies of GluA1–4 subunits. In CA1 pyramidal neurons ~80% of synaptic AMPARs are GluA1/A2 heteromers and the remaining ~20% are GluA2/A3 heteromers (Lu et al., 2009). GluA2-containing AMPARs exhibit a near linear I/V relationship whereas AMPARs lacking GluA2 are Ca^2+^ permeable and display marked inward rectification. We performed rectification assays to determine if our genetic manipulations produced insertion of GluA2-lacking AMPARs into CA3-CA1 synapses. Neither Rac1 overexpression nor Rac1 C18Y expression altered AMPAR-eEPSC rectification, and thus these genetic manipulations do not alter the GluA2 content of synaptic AMPARs (Figure 2F). Next, we wanted to determine if Rac1 overexpression or Rac1 C18Y expression alter the GluA1/A2 to GluA2/A3 AMPAR ratio at glutamatergic synapses. GluA1/A2 AMPARs exhibit slower decay kinetics relative to GluA2/A3 AMPARs, thus a shift in the GluA1/A2 to GluA2/A3 AMPAR ratio would manifest as a change in AMPAR-eEPSC decay kinetics (Herring et al., 2013). Both Rac1 and Rac1 C18Y transfected neurons showed the same decay kinetics as that of control neurons (Figure 2G). Thus, synaptic AMPAR subunit composition does not change in response to our genetic manipulations.

The selective alterations in AMPAR-eEPSC amplitudes produced by the overexpression of Rac1 or Rac1 C18Y expression could be due to a change in the relative number of AMPAR containing or AMPAR-lacking (“silent”) glutamatergic synapses (i.e., a change in quantal content). Alternatively, alterations in AMPAR-eEPSC amplitude could be due to a global change in the efficiency of AMPAR-mediated synaptic transmission across all functional synapses (a change in quantal size). To answer this question we used coefficient of variation analysis (CV analysis). CV analysis is presented as scatterplots with CV^−2^ values calculated for transfected cell/control cell pairs on the *y*-axis and mean eEPSC amplitude values of transfected cell/control cell pairs on the *x*-axis (Figure 2H). It has been shown theoretically and experimentally that changes in CV value are independent of quantal size but vary in a predictable manner with quantal content (del Castillo and Katz, 1954; Bekkers and Stevens, 1990; Malinow and Tsien, 1990). Thus, values approaching the horizontal line (*y* = 1) indicate a change in quantal size. In contrast, values close to the diagonal (*y* = *x*) line indicate a change in quantal content (Gray et al., 2011; Levy et al., 2015). Our CV analysis of Rac1 overexpression resulted in a linear regression line of the data falling closer to the horizontal line (Figure 2H). 95% confidence intervals of the data include the horizontal line but do not include the diagonal line. Therefore, we conclude that Rac1 overexpression-mediated increases in AMPAR-eEPSC amplitude largely arise from a uniform increase in AMPAR-mediated neurotransmission efficiency across all functional synapses. Interestingly, CV analysis of Rac1 C18Y data yielded average points that fall on the diagonal line and 95% confidence intervals that include the diagonal line and exclude the horizontal line (Figure 2H). These data indicate that Rac1 C18Y results in a reduction in the number of synapses that contain functional AMPARs. This could be due to reduction of synapse number or increase of AMPAR lacking synapses. We then used Structural Illumination Microscopy (SIM) to obtain super resolution images of dendritic spines from CA1 pyramidal neurons expressing GFP alone, GFP and Rac1 or GFP and Rac1 C18Y. We found neither Rac1 overexpression nor Rac1 C18Y expression resulted in a change in dendritic spine density (Figure 2I). Given that Rac1 C18Y expression does not affect NMDAR-eEPSC amplitude or PPF, these data suggest that Rac1 C18Y expression results in a potentially pathogenic increase in the number of AMPAR-lacking or “silent” glutamatergic synapses.

### Rac1 C18Y Prevents GTP-Mediated Activation of Rac1

Our modeling suggests that Rac1 C18Y will prevent GTP from activating Rac1. GTP associates with Rac1 resulting in Rac1 activation. Rac1 is able to inactivate itself through its ability to hydrolyze bound GTP, converting it to GDP. Guanine nucleotide exchange factors (GEFs) regulate Rac1 function by binding to Rac1 and removing GDP, allowing Rac1 to reassociate with GTP and reactivate. Thus, preventing Rac1’s ability to convert GTP to GDP renders Rac1 constitutively active. The only way to prevent activation of a constitutively active form of Rac1 is to directly interfere with GTP’s interaction with Rac1. If the C18Y mutation inhibits Rac1b function, this result will point to C18Y directly preventing a functional GTP interaction with Rac1.

**Figure 3:**
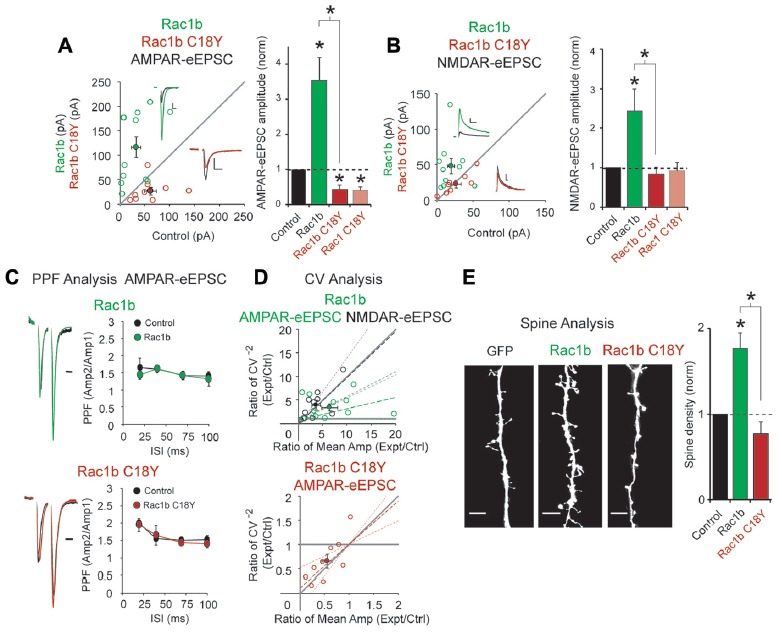
The ID-related mutation C18Y prevents synaptic potentiation by a constitutively active form of Rac1, Rac1b. **(A,B)** Scatterplots show eEPSC amplitudes for single pairs of CA1 pyramidal neurons transfected with Rac1b (green) or Rac1b C18Y (red) and their corresponding control neurons (*open circles*). Filled circles show mean ± SEM (*insets*). Current traces from control (*black*) and transfected *(green for Rac1b, red for Rac1b C18Y*) neurons are shown (*Scale* bars: 20 ms for AMPA, 50 ms for NMDA, 20 pA). Bar graphs show the average eEPSC amplitudes (± SEM) of neurons expressing Rac1b or Rac1b C18Y normalized to their respective average control eEPSC amplitudes. Wilcoxon Rank Sum Tests were used to compare across independent conditions (i.e., Rac1b and Rac1b C18Y in **(A,B)**, *P < 0.05). **(A)** Rac1b expression increased AMPAR-eEPSC amplitude (*n* = 12 pairs, *P < 0.05, Wilcoxon Signed Rank Test). Rac1b C18Y expression reduced AMPAR-eEPSC amplitude (*n* = 9 pairs, *P < 0.05, Wilcoxon Signed Rank Test). **(B)** Rac1b expression increased NMDAR-eEPSC amplitude (*n* = 10 pairs, *P < 0.05, Wilcoxon Signed Rank Test). Rac1b C18Y expression did not affect NMDAR-eEPSC amplitude (*n* = 8 pairs, *P* > 0.05, Wilcoxon Signed Rank Test). **(C)** Rac1b and Rac1b C18Y expression did not affect PPF ratios at interstimulus intervals of 20, 40, 70 and 100 ms (Upper plot: 20 ms: *n* = 6 pairs, 40 ms: *n* = 8 pairs, 70 ms: *n* = 4 pairs, 100 ms: *n* = 6 pairs, *P* > 0.05, Student’s *t*-test; Lower plot: 20 ms: *n* = 6 pairs, 40 ms: *n* = 8 pairs, 70 ms: *n* = 5 pairs, 100 ms: *n* = 6 pairs, P > 0.05, Student’s *t*-test). Peak 1-scaled current traces from control (*black*) and transfected (*green for Rac1b, red for Rac1b C18Y*) neurons are shown. (Scale bar: 20 ms). n.s., not significant. **(D)** CV analysis of AMPAR-eEPSC from pairs of control and Rac1b/Rac1b C18Y neurons and of NMDAR-eEPSCs from pairs of control and Rac1b neurons. CV^−2^ ratios are graphed against the mean amplitude ratio for each pair (*open circles*) (*green for Rac1b AMPAR-eEPSC*: *n* = 12 pairs; *black for Rac1b NMDAR-eEPSC*: *n* = 10 pairs; *red for Rac1b C18Y AMPAR-eEPSC*: *n* = 9 pairs). Filled circles show mean ± SEM. Dashed lines show linear regression and 95% confidence intervals. **(E)** Rac1b expression but not Rac1b C18Y expression increased dendritic spine density. Representative dendritic spine images from neurons transfected with GFP (control), Rac1b, or Rac1b C18Y are shown on the left (Scale bars: 5 μm). The bar graph shows average spine density (mean ± SEM) of neurons expressing Rac1b (control: 0.17 ± 0.015 spines/μM, *n* = 11; Rac1b: 0.31 ± 0.031 spines/μM, *n* = 10 pairs, *P < 0.05, Student’s *t*-test) or Rac1b C18Y (control: 0.24 ± 0.075 spines/μM, *n* = 7; Rac1b C18Y: 0.19 ± 0.033 spines/μM, *n* = 13, *P* > 0.05, Student’s *t*-test) normalized and compared to GFP expressing control neurons. Wilcoxon Rank Sum Test was used to compare across independent conditions (i.e., Rac1b and Rac1b C18Y in E, *P < 0.05).

We expressed Rac1b, a constitutively active form of Rac1 (Singh et al., 2004), and Rac1b C18Y in CA1 pyramidal neurons for 3 days. Consistent with its constitutively active feature, Rac1b expression led to a 4-fold increase in AMPAR-eEPSC amplitude, nearly twice that seen with wild-type Rac1 (Figures 2C, 3A). Rac1b also resulted in a 2.5-fold increase in NMDAR-eEPSCs, an effect that was not seen with wild-type Rac1 (Figures 2D, 3B). If C18Y prevents GTP from activating Rac1b, expression of Rac1b C18Y should prevent the constitutive activation and cause Rac1b C18Y and Rac1 C18Y to become functionally equivalent. We found that expression of Rac1b C18Y phenocopied Rac1 C18Y, producing a ~60% reduction in AMPAR-eEPSC amplitude compared to control neurons with no effect on NMDAR-eEPSC amplitude (Figures 3A,B). We also found that Rac1b and Rac1b C18Y overexpression do not alter PPF, indicating that neither manipulation modified presynaptic neurotransmitter release (Figure 3C). We then assessed the cause of alteration in AMPAR-eEPSCs and NMDAR-eEPSCs following Rac1b and Rac1b C18Y expression. CV analysis suggests that Rac1b, like Rac1, increases AMPAR function at all functional gluatamatergic synapses (Figure 3D). Rac1b C18Y, like Rac1 C18Y, inhibits AMPAR-eEPSC amplitude by reducing the number of glutamatergic synapses that contain functional AMPARs (Figure 3D). CV analysis of increased NMDAR-eEPSC amplitude following Rac1b expression results from an increase in quantal content, suggesting an increase in the number of synapses that express NMDARs (Figure 3E). Consistent with this finding, changes in NMDAR-eEPSCs commonly coincide with changes in glutamatergic synapse number. Because of this, we examined dendritic spine density following Rac1b expression. We found that Rac1b expression produces a nearly 2-fold increase in dendritic spine density. This increase accounts for the bulk of the NMDAR phenotype. Rac1b C18Y on the other hand prevented the synaptogenic effects of Rac1b (Figure 3E). Taken together, our data show that Rac1b C18Y prevents Rac1b’s effects on synaptic transmission and largely phenocopies Rac1 C18Y. Thus, we conclude that C18Y mutation prevents GTP from activating Rac1.

### Rac1 C18Y Prevents LTP Induction

**Figure 4:**
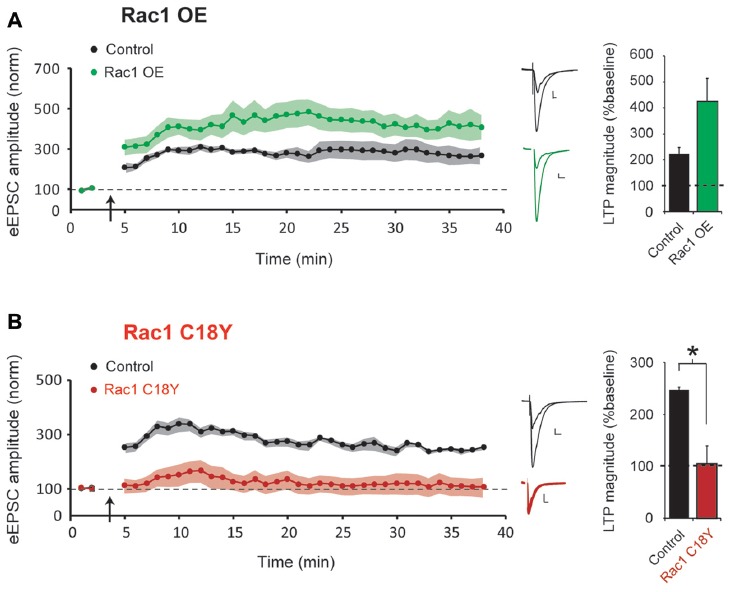
Rac1 C18Y expression prevents long-term potentiation (LTP) induction. **(A,B)** Plots of AMPAR-eEPSC amplitude of untransfected neurons (black) and neurons transfected with Rac1 (green) or Rac1 C18Y (red) normalized to the mean AMPAR-eEPSC amplitude before LTP induction (arrow). Sample AMPAR-eEPSC current traces from control (*black*) and transfected *(green for Rac1, red for Rac1 C18Y*) neurons before and after LTP inductions are shown (*Scale* bars: 20 ms, 20 pA). The bar graph shows individual LTP magnitude (mean ± SEM), Wilcoxon Signed Rank Test. Wilcoxon Rank Sum Test was used to compare across independent conditions (i.e., GFP and Rac1 C18Y in **(B)**, *P < 0.05). **(A)** Wild-type Rac1 expression in CA1 pyramidal neurons does not significantly affect LTP induction (control: *n* = 7 neurons; Rac1: *n* = 6 neurons). **(B)** Rac1 C18Y prevents LTP induction (control: *n* = 8 neurons; Rac1 C18Y: *n* = 6 neurons).

LTP is required for learning and memory formation in the brain. Rac1 activation has been implicated in LTP, and we have recently shown that the Rac1 GEFs Kalirin and Trio are critical for LTP induction (Herring and Nicoll, 2016b). LTP results in glutamatergic synapse unsilencing (Kerchner and Nicoll, 2008), and here we find that expression of a severe ID-related mutation in Rac1 results in an increased number of silent synapses. Thus, we were interested in whether this ID-related mutation in Rac1 influences the induction of LTP. To answer this question, *in utero* electroporation of embryonic day (E) 15 mice was used to express either Rac1 or Rac1 C18Y in hippocampal CA1 pyramidal neurons during early periods of brain development. Acute hippocampal slices were prepared from juvenile mice (postnatal day (P) 21-29 mice). We then used a “pairing” LTP induction protocol (see “Materials and Methods” section) that produces robust NMDAR-dependent LTP to examine LTP induction in CA1 pyramidal neurons overexpressing Rac1 or expressing Rac1 C18Y. We found that overexpression of wild-type Rac1 produced a modest but not statistically significant increase of LTP compared to untransfected control neurons (Figure 4A). In contrast, we found that expression of Rac1 C18Y abolished LTP induction (Figure 4B). Given the importance of LTP in information storage in the brain, our results suggest that Rac1 C18Y’s ability to suppress LTP induction likely contributes to the severe ID of this individual.

## Discussion

Accumulating evidence points to glutamatergic synapse dysregulation and Rac1-mediated actin polymerization as convergence points of a number of pathways implicated in ASD/ID (Joensuu et al., 2018). Rac1 is directly involved in glutamatergic synapse function and plasticity. However, the impact of ASD/ID-related mutations in Rac1 on glutamatergic synapses has not been investigated. In this study, we characterize the impact of a severe ID-related mutation in Rac1, C18Y, on glutamatergic synapse function. Our modeling shows that Rac1 C18Y likely prevents GTP activation of Rac1. Our electrophysiological data in hippocampal slices show that wild-type Rac1 overexpression selectively increases AMPAR-mediated neurotransmission across all functional glutamatergic synapses. This increase may be explained by either the insertion of additional AMPARs into synapses or modification of existing synaptic AMPARs. In contrast, we found that Rac1 C18Y expression reduces AMPAR-eEPSC amplitude compared to control neurons. The impact of Rac1 and Rac1 C18Y transfection on neurons did not differ across synaptic developmental time points and these manipulations did not affect spine density or presynaptic release. CV analysis revealed that reductions in AMPAR-mediated neurotransmission caused by Rac1 C18Y expression result from a reduction in the number of glutamatergic synapses that contain functional AMPARs. We also found that the C18Y mutation prevents the activity of a constitutively active Rac1, strongly suggesting that the C18Y mutation prevents GTP activation of Rac1. This finding is consistent with our mutational modeling. The precise mechanism behind Rac1 C18Y’s ability to increase the number of AMPAR-lacking synapses in neurons is unclear. We believe this effect most likely arises from either a dilution of endogenous wild-type synaptic Rac1 with a nonfunctioning form of Rac1 or the ability of Rac1 C18Y to bind to Rac1-activating GEF proteins and reduce their availability to bind to and activate functional Rac1 molecules. The individual harboring the Rac1 C18Y mutation is heterozygous for the mutation and, as a result, cells express both wild-type Rac1 and Rac1 C18Y. Thus, potential methods of competition between wild-type Rac1 and Rac1 C18Y are relevant.

Rac1 has been implicated in the induction of LTP, the cellular basis for learning and memory formation (Haditsch et al., 2009; Rex et al., 2009). We have recently found that two Rac1-activating GEF proteins, Kalirin and Trio, are required for CaMKII-dependent LTP induction (Herring and Nicoll, 2016b). Increases in Rac1 activity in dendritic spines are thought to promote actin-mediated structural changes of synapses that underlie synaptic AMPAR insertion during LTP (Herring and Nicoll, 2016a). We reasoned that this intellectual disability-related C18Y mutation in Rac1 may prevent Kalirin and Trio-mediated upregulation of Rac1 activity during LTP and thus inhibit LTP induction. Indeed, we find that in marked contrast to wild-type Rac1 overexpression, neuronal Rac1 C18Y expression prevents the induction of LTP. It is therefore likely that Rac1 C18Y’s potent ability to inhibit the induction of LTP contributes to the ID observed in this individual.

Synaptic Rac1 dysregulation is now believed to contribute to ASD/ID-related behavioral phenotypes in a number of established animal models of ASD/ID (Joensuu et al., 2018). Shank3 knockout mice, for example, are a well-established model for ASD/ID that display ASD/ID associated behaviors and exhibit pronounced synaptic dysfunction. A recent study showed that enhancing Rac1 function through the inhibition of a negative regulator of Rac1 activation alleviated both the synaptic and ASD-related behavioral phenotypes of these mice (Duffney et al., 2015). Fragile X syndrome, the most common inherited form of Autism, may also stem from synaptic Rac1 dysregulation. Fragile X syndrome is caused by the reduced expression of FMRP, a protein translation repressor protein encoded by the *Fmr1* gene. *Fmr1* knockout mice exhibit increased glutamatergic synapse density as well as behaviors that are similar to the human condition (Comery et al., 1997). These mice were found to have increased Rac1 expression (Bongmba et al., 2011). It was recently shown that inhibiting the Rac1 effector protein, PAK, reversed both the synaptic and behavioral phenotypes observed in *Fmr1* knockout mice (Dolan et al., 2013). Taken together, such evidence suggests that both Rac1 hypo and hyperfunction are responsible for pathological conditions at synapses that give rise to ASD/ID-related disorders. Consistent with this idea, we have recently discovered an ASD/ID-related *de novo* mutation hotspot in the Rac1 activation domain of Trio. Mutations in this domain either reduce or increase Trio’s ability to activate Rac1 (Sadybekov et al., 2017). Ultimately, dysregulation of synaptic Rac1 activity levels in either direction is likely to produce disruption of important regulatory mechanisms at glutamatergic synapses. For example, Rac1 is an important regulator of Cyfip1 function, with elevated Rac1 activation levels triggering a shift from Cyfip1’s involvement in translational regulation toward a more direct role in synaptic actin regulation (De Rubeis et al., 2013). In the present study we find that a Rac1 mutation in an individual with severe ID results in a synaptic phenotype nearly identical to that observed with hypofunctional ASD-related mutations in Trio. Thus, the Trio-Rac1 pathway may be a promising candidate for convergence of a number of ASD/ID associated factors, given that pathological mutations in the Trio-Rac1 axis of synaptic regulation produce a diverse array of glutamatergic synapse phenotypes that are similar to the varied synaptic phenotypes observed in animal models of ASD/ID. It will be necessary to determine the prevalence of altered Trio-Rac1 pathway function in patients with ASD/ID and establish whether Trio/Rac1-related therapies can be applied to reverse ASD/ID-related behavioral phenotypes in patients with these disorders.

## Author Contributions

CT and YK collected and analyzed the data. AS performed the modeling. SR provided professional support for data analysis. CT, YK, BH and VK wrote the article.

## Conflict of Interest Statement

The authors declare that the research was conducted in the absence of any commercial or financial relationships that could be construed as a potential conflict of interest.

## References

[B1] Bagni C. Greenough W. T. (2005). From mRNP trafficking to spine dysmorphogenesis: the roots of fragile X syndrome. Nat. Rev. Neurosci. 6, 376–387. 10.1038/nrn1667 15861180

[B2] Bekkers J. M. Stevens C. F. (1990). Presynaptic mechanism for long-term potentiation in the hippocampus. Nature 346, 724–729. 10.1038/346724a0 2167454

[B3] Bongmba O. Martinez L. Elhardt M. Butler K. Tejada-Simon M. (2011). Modulation of dendritic spines and synaptic function by Rac1: a possible link to Fragile X syndrome pathology. Brain Res. 1399, 79–95. 10.1016/j.brainres.2011.05.020 21645877 PMC3131096

[B4] Comery T. A. Harris J. B. Willems P. J. Oostra B. A. Irwin S. A. Weiler I. J.. (1997). Abnormal dendritic spines in fragile X knockout mice: maturation and pruning deficits. Proc. Natl. Acad. Sci. U S A 94, 5401–5404. 10.1073/pnas.94.10.5401 9144249 PMC24690

[B6] del Castillo J. Katz B. (1954). Quantal components of the end-plate potential. J. Physiol. 124, 560–573. 10.1113/jphysiol.1954.sp005129 13175199 PMC1366292

[B5] De Rubeis S. Pasciuto E. Li K. W. Fernández E. Di Marino D. Buzzi A.. (2013). CYFIP1 coordinates mRNA translation and cytoskeleton remodeling to ensure proper dendritic spine formation. Neuron 79, 1169–1182. 10.1016/j.neuron.2013.06.039 24050404 PMC3781321

[B7] Dolan B. Duron S. Campbell D. Vollrath B. Shankaranarayana Rao B. S. Ko H.-Y.. (2013). Rescue of fragile X syndrome phenotypes in Fmr1 KO mice by the small-molecule PAK inhibitor FRAX486. Proc. Natl. Acad. Sci. U S A 110, 5671–5676. 10.1073/pnas.1219383110 23509247 PMC3619302

[B8] Duffney L. J. Zhong P. Wei J. Matas E. Cheng J. Qin L.. (2015). Autism-like deficits in shank3-deficient mice are rescued by targeting actin regulators. Cell Rep. 11, 1400–1413. 10.1016/j.celrep.2015.04.064 26027926 PMC4464902

[B10] Gray J. A. Shi Y. Usui H. During M. J. Sakimura K. (2011). Distinct modes of AMPA receptor suppression at developing synapses by GluN2A and GluN2B: single-cell NMDA receptor subunit deletion *in vivo*. Neuron 71, 1085–1101. 10.1016/j.neuron.2011.08.007 21943605 PMC3183990

[B11] Haditsch U. Leone D. P. Farinelli M. Chrostek-Grashoff A. Brakebusch C. Mansuy I. M.. (2009). A central role for the small GTPase Rac1 in hippocampal plasticity and spatial learning and memory. Mol. Cell. Neurosci. 41, 409–419. 10.1016/j.mcn.2009.04.005 19394428 PMC2705331

[B12] Herring B. E. Nicoll R. A. (2016a). Long-term potentiation: from CaMKII to AMPA receptor trafficking. Annu. Rev. Physiol. 78, 351–365. 10.1146/annurev-physiol-021014-071753 26863325

[B14] Herring B. E. Nicoll R. A. (2016b). Kalirin and Trio proteins serve critical roles in excitatory synaptic transmission and LTP. Proc. Natl. Acad. Sci. U S A 113, 2264–2269. 10.1073/pnas.1600179113 26858404 PMC4776457

[B13] Herring B. E. Shi Y. Suh Y. H. Zheng C.-Y. Y. Blankenship S. M. Roche K. W.. (2013). Cornichon proteins determine the subunit composition of synaptic AMPA receptors. Neuron 77, 1083–1096. 10.1016/j.neuron.2013.01.017 23522044 PMC3652566

[B15] Hirshberg M. Stockley R. W. Dodson G. Webb M. R. (1997). The crystal structure of human rac1, a member of the rho-family complexed with a GTP analogue. Nat. Struct. Biol. 4, 147–152. 10.1038/nsb0297-147 9033596

[B16] Joensuu M. Lanoue V. Hotulainen P. (2018). Dendritic spine actin cytoskeleton in autism spectrum disorder. Prog. Neuropsychopharmacol. Biol. Psychiatry 84, 362–381. 10.1016/j.pnpbp.2017.08.023 28870634

[B17] Ke M. T. Fujimoto S. Imai T. (2013). SeeDB: a simple and morphology-preserving optical clearing agent for neuronal circuit reconstruction. Nat. Neurosci. 16, 1154–1161. 10.1038/nn.3447 23792946

[B18] Kerchner G. A. Nicoll R. A. (2008). Silent synapses and the emergence of a postsynaptic mechanism for LTP. Nat. Rev. Neurosci. 9, 813–825. 10.1038/nrn2501 18854855 PMC2819160

[B19] Lek M. Karczewski K. J. Minikel E. V. Samocha K. E. Banks E. Fennell T.. (2016). Analysis of protein-coding genetic variation in 60,706 humans. Nature 536, 285–291. 10.1038/nature19057 27535533 PMC5018207

[B20] Lelieveld S. H. Reijnders M. R. Pfundt R. Yntema H. G. Kamsteeg E.-J. de Vries P.. (2016). Meta-analysis of 2,104 trios provides support for 10 new genes for intellectual disability. Nat. Neurosci. 19, 1194–1196. 10.1038/nn.4352 27479843

[B21] Levy J. M. Chen X. Reese T. S. Nicoll R. A. (2015). Synaptic consolidation normalizes AMPAR quantal size following MAGUK loss. Neuron 87, 534–548. 10.1016/j.neuron.2015.07.015 26247861 PMC4596923

[B22] Lu W. Shi Y. Jackson A. Bjorgan K. During M. J. Sprengel R.. (2009). Subunit composition of synaptic AMPA receptors revealed by a single-cell genetic approach. Neuron 62, 254–268. 10.1016/j.neuron.2009.02.027 19409270 PMC3632349

[B23] Malinow R. Tsien R. W. (1990). Presynaptic enhancement shown by whole-cell recordings of long-term potentiation in hippocampal slices. Nature 346, 177–180. 10.1038/346177a0 2164158

[B24] Martinez L. A. Tejada-Simon M. V. (2011). Pharmacological inactivation of the small GTPase Rac1 impairs long-term plasticity in the mouse hippocampus. Neuropharmacology 61, 305–312. 10.1016/j.neuropharm.2011.04.017 21569781 PMC3106418

[B25] Matsuzaki M. Honkura N. Ellis-Davies G. C. R. Kasai H. (2004). Structural basis of long-term potentiation in single dendritic spines. Nature 429, 761–766. 10.1038/nature02617 15190253 PMC4158816

[B9] Mefford H. C. Batshaw M. L. Hoffman E. P. (2012). Genomics, intellectual disability, and autism. N. Engl. J. Med. 366, 733–743. 10.1056/NEJMra1114194 22356326 PMC4107681

[B26] Pavlowsky A. Chelly J. Billuart P. (2012). Major synaptic signaling pathways involved in intellectual disability. Mol. Psychiatry 17:663. 10.1038/mp.2012.79 22722779

[B27] Rex C. S. Chen L. Y. Sharma A. Liu J. Babayan A. H. Gall C. M.. (2009). Different Rho GTPase-dependent signaling pathways initiate sequential steps in the consolidation of long-term potentiation. J. Cell Biol. 186, 85–97. 10.1083/jcb.200901084 19596849 PMC2712993

[B51] Singh A. Karnoub A. Palmby T. Lengyel E. Sondek J. Der C. (2004). Rac1b, a tumor associated, constitutively active Rac1 splice variant, promotes cellular transformation. Oncogene. 23, 9369–9380. 10.1038/sj.onc.1208182 15516977

[B28] Sadybekov A. Tian C. Arnesano C. Katritch V. Herring B. E. (2017). An autism spectrum disorder-related *de novo* mutation hotspot discovered in the GEF1 domain of Trio. Nat. Commun. 8:601. 10.1038/s41467-017-00472-0 28928363 PMC5605661

[B29] Schenck A. Bardoni B. Langmann C. Harden N. Mandel J. L. Giangrande A. (2003). CYFIP/Sra-1 controls neuronal connectivity in *Drosophila* and links the Rac1 GTPase pathway to the fragile X protein. Neuron 38, 887–898. 10.1016/s0896-6273(03)00354-4 12818175

[B30] Schnell E. Sizemore M. Karimzadegan S. Chen L. Bredt D. Nicoll R. (2002). Direct interactions between PSD-95 and stargazin control synaptic AMPA receptor number. Proc. Natl. Acad. Sci. U S A 99, 13902–13907. 10.1073/pnas.172511199 12359873 PMC129795

[B31] Stoppini L. Buchs P.-A. Muller D. (1991). A simple method for organotypic cultures of nervous tissue. J. Neurosci. Methods 37, 173–182. 10.1016/0165-0270(91)90128-m 1715499

[B32] Tashiro A. Minden A. Yuste R. (2000). Regulation of dendritic spine morphology by the rho family of small GTPases: antagonistic roles of Rac and Rho. Cereb. Cortex 10, 927–938. 10.1093/cercor/10.10.927 11007543

[B33] Volk L. Chiu S.-L. Sharma K. Huganir R. (2014). Glutamate synapses in human cognitive disorders. Annu. Rev. Neurosci. 38, 127–149. 10.1146/annurev-neuro-071714-033821 25897873

[B34] Wiens K. M. Lin H. Liao D. (2005). Rac1 induces the clustering of AMPA receptors during spinogenesis. J. Neurosci. 25, 10627–10636. 10.1523/JNEUROSCI.1947-05.2005 16291935 PMC6725855

[B35] Worthylake D. K. Rossman K. L. Sondek J. (2000). Crystal structure of Rac1 in complex with the guanine nucleotide exchange region of Tiam1. Nature 408, 682–688. 10.1038/35047014 11130063

[B50] Xiang Z. Greenwood A. C. Kairiss E. W. Brown T. H. (1994). Quantal mechanism of long-term potentiation in hippocampal mossy-fiber synapses. J. Neurophysiol. 71, 2552–2556. 10.1152/jn.1994.71.6.2552 7931534

[B36] Zeidán-Chuliá F. Rybarczyk-Filho J. L. Salmina A. B. de Oliveira B.-H. Noda M. Moreira J. C. (2013). Exploring the multifactorial nature of autism through computational systems biology: calcium and the Rho GTPase RAC1 under the spotlight. Neuromolecular Med. 15, 364–383. 10.1007/s12017-013-8224-3 23456597

[B37] Zoghbi H. Y. Bear M. F. (2012). Synaptic dysfunction in neurodevelopmental disorders associated with autism and intellectual disabilities. Cold Spring Harb. Perspect. Biol. 4:a009886. 10.1101/cshperspect.a009886 22258914 PMC3282414

