# Supplementary material for: An Intellectual Disability-Related Missense Mutation in Rac1 Prevents LTP Induction
Source: Front Mol Neurosci. 2018 Jul 6;11:223. doi: 10.3389/fnmol.2018.00223 (PMC6049044; doi:10.3389/fnmol.2018.00223)
Supplement: Supplementary file 1 [file Image_1.pdf]

Supplementary Material

An Intellectual Disability-Related Missense Mutation in Rac1 Prevents LTP Induction

Chen Tian, Yuni Kay, Anastasiia Sadybekov, Sadhna Rao, Vsevolod Katritch, Bruce Herring*

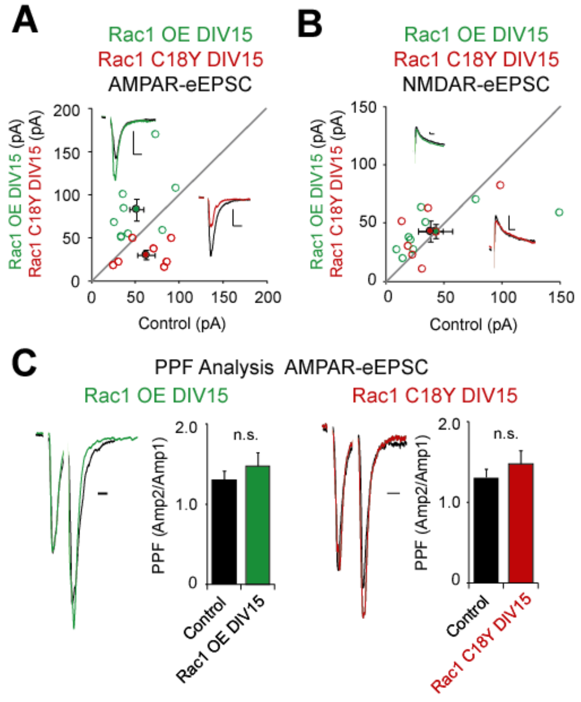

FIGURE S1 | Rac1 C18Y weakens glutamatergic synaptic transmission in DIV15 slices, related to Figure 2. **(A, B)** Scatterplots show eEPSC amplitudes recorded at DIV15 for single pairs of CA1 pyramidal neurons transfected with Rac1 (green) or Rac1 C18Y (red) and their corresponding control neurons (open circles). Filled circles show mean ± SEM (insets). Current traces from control (black) and transfected (green for Rac1, red for Rac1 C18Y) neurons are shown (Scale bars: 20 ms for AMPA, 50 ms for NMDA, 20pA). Bar graph and n for each condition are included in Figure 2. **(C)** Rac1 and Rac1 C18Y expression did not affect paired-pulse facilitation (PPF) ratios at inter stimulus intervals of 40 ms (Rac1: n = 7 pairs, P > 0.05, Student's t-test; Rac1 C18Y: n = 6 pairs, P > 0.05, Student's t-test). Peak 1-scaled current traces from control (black) and transfected (green for Rac1, red for Rac1 C18Y) neurons are shown. (Scale bar: 20 ms). n.s., not significant.
